# Patient and tumour characteristics associated with inclusion in Cancer patient pathways in Norway in 2015–2016

**DOI:** 10.1186/s12885-020-06979-y

**Published:** 2020-05-30

**Authors:** Yngvar Nilssen, Odd Terje Brustugun, Morten Tandberg Eriksen, Erik Skaaheim Haug, Bjørn Naume, Bjørn Møller

**Affiliations:** 1 Department of Registration, Cancer Registry of Norway, Oslo, Norway; 2 Section of Oncology, Drammen Hospital, Vestre Viken Hospital Trust, Drammen, Norway; 3 Division of Surgery, Inflammatory Diseases and Transplantation, Oslo University Hospital, Oslo, Norway; 4 Institute of Clinical Medicine, University of Oslo, Oslo, Norway; 5 Section of Urology, Vestfold Hospital Trust, Tønsberg, Norway; 6 Institute of Cancer Genomics and Informatics, Oslo University Hospital, Oslo, Norway; 7 Department of Oncology, Oslo University Hospital, Oslo, Norway

**Keywords:** Cancer patient pathways, Colorectal cancer, Lung cancer, Breast cancer, Prostate cancer

## Abstract

**Background:**

Cancer patient pathways (CPPs) were implemented in 2015 to reduce waiting time, regional variation in waiting time, and to increase the predictability of cancer care for the patients. The aims of this study were to see if the national target of 70% of all cancer patients being included in a CPP was met, and to identify factors associated with CPP inclusion.

**Methods:**

All patients registered with a colorectal, lung, breast or prostate cancer diagnosis at the Cancer Registry of Norway in the period 2015–2016 were linked with the Norwegian Patient Registry for CPP information and with Statistics Norway for sociodemographic variables. Multivariable logistic regression examined if the odds of not being included in a CPP were associated with year of diagnosis, age, sex, tumour stage, marital status, education, income, region of residence and comorbidity.

**Results:**

From 2015 to 2016, 30,747 patients were diagnosed with colorectal, lung, breast or prostate cancer, of whom 24,429 (79.5%) were included in a CPP. Significant increases in the probability of being included in a CPP were observed for colorectal (79.1 to 86.2%), lung (79.0 to 87.3%), breast (91.5 to 97.2%) and prostate cancer (62.2 to 76.2%) patients (p < 0.001). Increasing age was associated with an increased odds of not being included in a CPP for lung (p < 0.001) and prostate cancer (p < 0.001) patients. Colorectal cancer patients < 50 years of age had a two-fold increase (OR = 2.23, 95% CI: 1.70–2.91) in the odds of not being included in a CPP. The odds of no CPP inclusion were significantly increased for low income colorectal (OR = 1.24, 95%CI: 1.00–1.54) and lung (OR = 1.52, 95%CI: 1.16–1.99) cancer patients. Region of residence was significantly associated with CPP inclusion (p < 0.001) and the probability, adjusted for case-mix ranged from 62.4% in region West among prostate cancer patients to 97.6% in region North among breast cancer patients.

**Conclusions:**

The national target of 70% was met within 1 year of CPP implementation in Norway. Although all patients should have equal access to CPPs, a prostate cancer diagnosis, older age, high level of comorbidity or low income were significantly associated with an increased odds of not being included in a CPP.

## Background

Time to cancer diagnosis, time to treatment and survival rates vary within and between countries. In order to reduce unnecessary and clinically unjustifiable delays, which may be attributable to the patient, doctor or system, several initiatives were implemented across Europe over the last two decades [1–5]. In 2000, the United Kingdom implemented urgent referral pathways and in 2005, the Catalonian Health Service in Spain launched the Cancer Fast-track Programme [6, 7]. The aim of both programs was to reduce the time that elapsed between suspicion of cancer and the start of initial treatment. In the period 2007–2008 Denmark implemented cancer patients pathways (CPPs) to reduce waiting times and regional variation, and to improve cancer survival [8, 9]. The Danish initiative strongly influenced the implementation of CPPs in both Sweden in 2015–2018 and Norway in 2015 [10, 11].

In the period 2007–2016, a significant reduction in waiting time to surgery and radiotherapy was seen for the four largest cancer sites in Norway [12]. While CPPs were implemented to reduce waiting times and regional variation, the need for predictability of the cancer care for patients and their relatives was strongly emphasised. As in Denmark and Sweden, CPPs in Norway can be described as a set of maximum days that patients should wait from a hospital referral to the first specialist visit, to a clinical decision and finally to the start of treatment. The number of days varies between the different cancer pathways [13–16]. A patient should be referred to a CPP if the doctor has a “reasonable suspicion of cancer” based on the patient’s symptoms. A patient can further be referred to a CPP by three different sources: GP, specialist or hospital. For a patient to be included in a CPP, the referral must be labelled as “cancer patient pathway”. The patients who are included in a CPP are assigned a cancer pathway coordinator who becomes their primary contact in the health system. In Norway, the national aim is to have a minimum of 70% of all cancer patients included in a CPP [17–20].

Norway has a free, national health care system that should be equally available to every citizen independent of personal characteristics, social status and area of residence. However, data published by the Norwegian Directorate of Health have shown substantial geographical variation in the proportion of cancer patients being referred to a CPP [21]. Therefore, the aims of this paper were to evaluate the inclusion of patients diagnosed with colorectal, lung, breast or prostate cancer in a CPP the first 2 years after implementation in Norway, and whether any patient- or tumour-related factors were associated with being registered as a CPP patient.

## Methods

### Cancer registry of Norway

Since 1953, it has been mandatory for all hospitals, pathology laboratories and general practitioners (GP) in Norway to report all newly diagnosed malignant disease to the Cancer Registry of Norway (CRN). The CRN also receives death certificates for all patients with a cancer diagnosis from the Cause of Death Registry. Using the personal identification number assigned to all Norwegian citizens since 1964, the CRN is linked monthly with the National Population Register to update vital status (death or emigration), and three times per year with the Norwegian Patient Registry (NPR) to ensure completeness of cancer cases. The quality, comparability, completeness, validity, and timeliness of the data in the CRN have been evaluated to be high, with an estimated completeness of 98.8% for all cancer sites together [22].

### Norwegian patient registry

The NPR is a national health register that holds data on all patient visits to government-funded hospitals in Norway. Reporting to the NPR is mandatory, and its database covers over 99% of all patient visits to specialised health care services [23]. These also include data regarding CPPs. From 2008 the NPR data also include personal identification numbers, thus enabling researchers and health authorities to follow the disease trajectory of patients between different sectors and hospitals.

### Statistics Norway

The national statistics institute, Statistics Norway, holds individual-level information in areas such as population, health, finance and education for the entire Norwegian population. Education data have been collected from various national databases since 1970. The tax authorities provide Statistics Norway with personal and household income data, which are available from 1967 and 1993, respectively, while information about type of household is available from 2004 onwards.

### Data linkage

The study population included all patients with a colorectal (ICD-10 code C18–20), lung (ICD-10 code C33–34), breast (ICD-10 code C50) or prostate (ICD-10 code C61) cancer diagnosis registered at the CRN between 1 January 2015 and 31 December 2016. Information from the NPR was linked to identify which patients were included in a CPP and the patient’s level of co-existing diseases (i.e., comorbidities). Information about the patient’s socioeconomic status (SES), measured through household income and education, was obtained from Statistics Norway.

### Study population

Between 1 January 2015 and 31 December 2016, 32,055 cases were identified with a primary colorectal, lung, breast or prostate cancer diagnosis. Cases registered based on either autopsy (n = 49) or death certificate alone (n = 389) were excluded. Also male breast cancer cases (n = 54), cases under 18 years of age (n = 11), cases with unknown place of residence (n = 260), unknown education (n = 221), unknown household income (n = 3) and unknown type of household (n = 321) were excluded from the analyses. As a result, 30,747 cases were eligible for analyses.

### Classification of variables

Date of diagnosis was defined as the date of the first histologically verified diagnosis registered at the CRN, which most often was based on a biopsy. The proportions of diagnoses morphologically verified (either histologically or cytologically) were 97.6% (colorectal), 90.2% (lung), 99.8% (breast) and 97.9% (prostate). Radiologically confirmed diagnoses without morphological confirmation represent < 1% of colorectal, breast and prostate cancer diagnoses and 6.1% of lung cancer diagnoses. For patients whose tumour was not morphologically verified, the date of diagnosis was set as the date from the clinical notification form.

### Stage

Stage of disease was categorised as localised, regional, metastatic, or unknown [24]. For breast cancer, stage was classified as stage I-II, III, IV or unknown. Stage I and II are grouped together since they follow the same treatment pathway and the distinction between these groups occur post-surgery based on tumour size or lymph node metastasis. Stage IV breast cancer may include synchronic, but not metachronic tumours, since by local CRN rules, appear after the diagnostic period of 4 months. For staging, notifications received within the diagnosis period at the CRN, defined as the month of diagnosis plus an additional 4 months, were used.

### Region

**Fig. 1:**
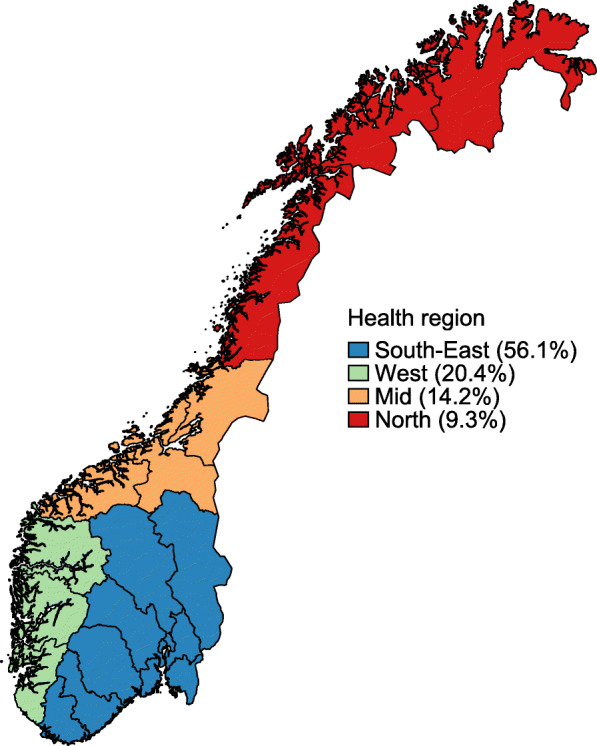
A map of the different health regions in Norway; South-East, West, Mid and North and the proportion of colorectal, lung, breast and prostate cancer patients diagnosed within each region

Norway consists of four health regions that are responsible for specialised health care in their catchment areas: South-East, West, Mid and North (Fig. 1). Regional affiliation was based on a patient’s place of residence at the time of diagnosis, independent of where the patient was diagnosed or treated.

### Socioeconomic (income, education) and marital status

A patient’s socioeconomic status was measured using individual information about household income and the highest level of obtained education. Household income included wages, self-employment capital income, pension and social benefits earned the year prior to diagnosis. The equivalised household income (square root scale), a measure adjusting for the number of people living in the household, was used and grouped as low, intermediate or high, based on the 20th and 80th sex-specific percentiles of household income in the entire cancer population [25]. Education was grouped as low (elementary school), intermediate (high school) or high (university). A patient’s marital status was categorised as single (registered as not married, widow, divorced or separated) or married (registered as married or partner).

### Comorbidity

A patient’s co-existing diseases were measured using a modified version of the Charlson Comorbidity Index (CCI) using diagnostic codes (ICD-10) from hospitalisations within 2 years prior to, and including, the date of diagnosis [26, 27]. A score was determined for each of a patient’s recorded co-existing diseases based on its severity, and the combination of these scores resulted in a modified CCI. The index was grouped into “no hospital admissions”, low (CCI = 0), intermediate (CCI = 1,2) or high (CCI = 3+).

### Statistical analysis

Pearson’s chi-squared test was used to assess differences between the categories of the explanatory variables and a dichotomous variable indicating whether a patient was included in a CPP. Multivariable logistic regressions, with not being included in a CPP as the dependent variable, were both performed for all cancer sites together and stratified for each cancer site, adjusted for case-mix, i.e., year of diagnosis, age group and stage at diagnosis, sex, region, income group, education group, marital status and comorbidity index [28]. Probabilities of being included in a CPP, adjusted for case-mix are presented in the supplementary material. Although colon and rectum cancer patients share the same CPP, a stratified analysis was performed to examine if differences between the two sites existed. Where the results differ between colon and rectum cancer patients, results are shown separately. Wald test was used to assess the significance of the different explanatory variables and potential interactions. A p-value < 0.05 was considered significant. The statistical program Stata 16.1 was used for all analyses [29].

## Results

**Fig. 2:**
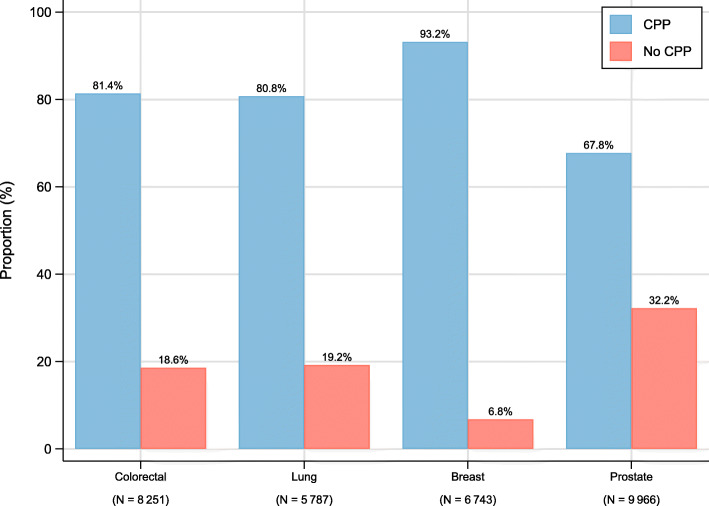
The proportion of all colorectal, lung, breast and prostate cancer patients included in a cancer patient pathway in 2015–2016 in Norway

Figure 2 shows the differences in proportions included in a CPP for each cancer type. In 2015–2016 the proportions of cases in a CPP ranged from 67.8% among prostate cancer to 93.2% for breast cancer, with colorectal and lung cancers being around 80.0%. Supplementary Table 1 shows the distribution of variables among all cancer patients, as well as, separately for patients who were, and were not, included in a CPP.

### Year of diagnosis

The proportion of colorectal, lung, breast and prostate cancer patients included in a CPP increased from 75.0% in 2015 to 83.9% in 2016 (Table 1). The cancer-specific increases ranged from 6.5 percentage points for breast cancer patients to 12.6 percentage points for prostate cancer patients (Supplementary Table S1). From February 2015 onwards, over 70% of patients with colorectal, lung or breast cancer were included in a CPP, while prostate cancer patients did not achieve this level until January 2016 (data not shown).

**Table 1:** Patient characteristics and proportion included in a cancer patient pathway for colorectal, lung, breast and prostate cancer patients diagnosed in 2015–2016 in Norway

	Colorectal		Lung		Breast		Prostate	
	N	% CPP	N	% CPP	N	% CPP	N	% CPP
**Year of diagnosis**								
2015	4133	77.7%	2901	76.6%	3409	90.0%	4971	61.5%
2016	4118	85.1%	2886	84.9%	3334	96.5%	4995	74.1%
**Age group**								
18–49	487	68.8%	135	84.4%	1282	94.5%	133	77.4%
50–59	906	83.8%	566	85.2%	1623	92.9%	1266	73.7%
60–69	2106	84.9%	1830	84.9%	2019	93.5%	4035	75.8%
70–79	2643	83.8%	2160	82.8%	1074	92.8%	3292	67.1%
80–89	1799	79.6%	999	68.6%	586	92.0%	1102	38.6%
90+	310	61.0%	97	51.5%	159	89.9%	138	16.7%
**Sex**								
Female	4034	80.3%	2769	80.0%	6743	93.2%		
Male	4217	82.4%	3018	81.5%			9966	67.8%
**Stage**^**a**^								
A	1779	78.7%	1056	90.7%	5394	94.4%	4793	63.7%
B	4128	86.4%	1621	87.9%	764	94.4%	2705	77.3%
C	1720	75.4%	2332	75.0%	233	76.4%	620	58.7%
D	624	72.4%	778	69.5%	352	83.5%	1848	67.3%
**Marital status**								
Single	3669	78.5%	2797	79.2%	3146	93.3%	2970	65.9%
Married	4582	83.7%	2990	82.2%	3597	93.2%	6996	68.6%
**Education**								
Low	2515	79.5%	2386	79.0%	1523	92.1%	2126	63.8%
Intermediate	3943	82.3%	2716	81.5%	3039	93.3%	5057	69.1%
High	1793	82.0%	685	84.1%	2181	93.9%	2783	68.4%
**Income**								
Low	1109	79.4%	912	75.8%	723	91.0%	800	59.8%
Intermediate	5180	81.2%	3951	80.7%	3318	92.6%	5800	66.5%
High	1962	83.2%	924	86.0%	2702	94.6%	3366	71.9%
**Region**								
South-East	4429	80.1%	3196	79.8%	3929	94.7%	5695	69.0%
West	1759	82.6%	1236	81.1%	1314	93.1%	1976	60.0%
Mid	1223	80.0%	747	77.9%	923	84.9%	1466	73.5%
North	840	87.7%	608	88.5%	577	96.9%	829	67.4%
**Comorbidity**								
No admissions	310	82.3%	90	90.0%	1482	94.7%	1188	59.1%
0	5949	82.4%	3144	82.7%	4599	93.0%	6960	71.7%
1–2	1675	79.5%	2132	79.5%	576	92.4%	1563	60.6%
3+	317	71.9%	421	71.0%	86	87.2%	255	43.9%

*Abbreviation*: *CPP* cancer patient pathways

^a^Colorectal, lung and prostate: Localised (A), Regional (B), Metastasis (C), Unknown (D). Breast: Stage I-II (A), Stage III (B), Stage IV (C), Unknown (D)

### Age group

There were statistically significant gradients, indicating higher odds of not being included in a CPP with increasing age, for colorectal (older than 90 years), lung (from 80 years and older) and prostate cancer (from 70 years and older). The likelihood of not being included in a CPP for colorectal cancer was increased for patients under 50 years of age (OR = 2.23, 95% CI: 1.70–2.91) (Table 2). The adjusted probability of CPP inclusion for colorectal cancer patients under 50 years was 69.9%, while the inclusion ranged from 81.7 to 85.2% for the other age groups (except 90+) (Supplementary Table 2). For breast cancer, there was no significant association between CPP and age group (p = 0.138).

**Table 2:** Univariable and multivariable analysis for not being included in cancer patient pathways among colorectal, lung, breast and prostate cancer patients diagnosed in 2015–2016 in Norway

	Colorectal		Lung		Breast		Prostate	
	Univariate	Multivariate	Univariate	Multivariate	Univariate	Multivariate	Univariate	Multivariate
	OR [95%CI]*	OR [95%CI]*	OR [95%CI]*	OR [95%CI]*	OR [95%CI]*	OR [95%CI]*	OR [95%CI]*	OR [95%CI]*
**Year of diagnosis**								
2015	1.00 [1.00,1.00]	1.00 [1.00,1.00]	1.00 [1.00,1.00]	1.00 [1.00,1.00]	1.00 [1.00,1.00]	1.00 [1.00,1.00]	1.00 [1.00,1.00]	1.00 [1.00,1.00]
2016	0.61 [0.55,0.69]	0.60 [0.54,0.68]	0.58 [0.51,0.66]	0.55 [0.47,0.63]	0.33 [0.27,0.41]	0.31 [0.25,0.39]	0.56 [0.51,0.61]	0.51 [0.47,0.56]
p-value	< 0.001	< 0.001	< 0.001	< 0.001	< 0.001	< 0.001	< 0.001	< 0.001
**Age group**								
18–49	2.34 [1.81,3.04]	2.23 [1.70,2.91]	1.06 [0.63,1.78]	1.18 [0.69,2.01]	0.75 [0.55,1.02]	0.77 [0.56,1.06]	0.82 [0.53,1.25]	0.75 [0.48,1.16]
50–59	1.00 [1.00,1.00]	1.00 [1.00,1.00]	1.00 [1.00,1.00]	1.00 [1.00,1.00]	1.00 [1.00,1.00]	1.00 [1.00,1.00]	1.00 [1.00,1.00]	1.00 [1.00,1.00]
60–69	0.92 [0.74,1.14]	0.89 [0.72,1.11]	1.02 [0.78,1.33]	1.00 [0.76,1.32]	0.91 [0.70,1.18]	0.88 [0.67,1.15]	0.89 [0.77,1.03]	0.94 [0.81,1.09]
70–79	1.00 [0.82,1.23]	0.93 [0.75,1.16]	1.19 [0.92,1.54]	1.11 [0.85,1.45]	1.00 [0.74,1.35]	0.79 [0.57,1.10]	1.37 [1.19,1.59]	1.58 [1.35,1.85]
80–89	1.32 [1.07,1.63]	1.15 [0.92,1.45]	2.63 [2.01,3.44]	2.21 [1.67,2.94]	1.13 [0.80,1.61]	0.74 [0.49,1.10]	4.46 [3.75,5.31]	5.59 [4.60,6.78]
90+	3.31 [2.48,4.41]	2.50 [1.83,3.40]	5.39 [3.40,8.55]	4.68 [2.87,7.63]	1.45 [0.84,2.52]	0.92 [0.50,1.68]	14.0 [8.8,22.3]	17.3[10.7,28.0]
p-value	< 0.001	< 0.001	< 0.001	< 0.001	0.138	0.509	< 0.001	< 0.001
**Sex**								
Female	1.00 [1.00,1.00]	1.00 [1.00,1.00]	1.00 [1.00,1.00]	1.00 [1.00,1.00]	1.00 [1.00,1.00]	1.00 [1.00,1.00]		
Male	0.87 [0.78,0.97]	0.93 [0.83,1.05]	0.91 [0.80,1.04]	0.86 [0.75,0.99]			1.00 [1.00,1.00]	1.00 [1.00,1.00]
p-value	0.014	0.240	0.152	0.040				
**Stage**^**a**^								
A	1.00 [1.00,1.00]	1.00 [1.00,1.00]	1.00 [1.00,1.00]	1.00 [1.00,1.00]	1.00 [1.00,1.00]	1.00 [1.00,1.00]	1.00 [1.00,1.00]	1.00 [1.00,1.00]
B	0.58 [0.50,0.67]	0.57 [0.49,0.66]	1.34 [1.04,1.74]	1.41 [1.09,1.83]	1.01 [0.72,1.40]	1.02 [0.73,1.43]	0.52 [0.46,0.57]	0.44 [0.39,0.49]
C	1.20 [1.03,1.41]	1.19 [1.01,1.40]	3.25 [2.59,4.08]	3.44 [2.72,4.35]	5.21 [3.77,7.20]	5.94 [4.20,8.40]	1.24 [1.04,1.47]	0.74 [0.61,0.89]
D	1.41 [1.14,1.73]	1.22 [0.98,1.52]	4.28 [3.31,5.54]	4.08 [3.12,5.32]	3.33 [2.45,4.51]	3.36 [2.41,4.68]	0.86 [0.76,0.96]	0.69 [0.61,0.78]
p-value	< 0.001	< 0.001	< 0.001	< 0.001	< 0.001	< 0.001	< 0.001	< 0.001
**Marital status**								
Single	1.00 [1.00,1.00]	1.00 [1.00,1.00]	1.00 [1.00,1.00]	1.00 [1.00,1.00]	1.00 [1.00,1.00]	1.00 [1.00,1.00]	1.00 [1.00,1.00]	1.00 [1.00,1.00]
Married	0.71 [0.64,0.79]	0.81 [0.72,0.92]	0.82 [0.72,0.94]	0.93 [0.81,1.08]	1.02 [0.84,1.23]	1.03 [0.83,1.28]	0.89 [0.81,0.97]	0.89 [0.80,0.98]
p-value	< 0.001	< 0.001	0.003	0.351	0.870	0.776	0.010	0.022
**Education**								
Low	1.00 [1.00,1.00]	1.00 [1.00,1.00]	1.00 [1.00,1.00]	1.00 [1.00,1.00]	1.00 [1.00,1.00]	1.00 [1.00,1.00]	1.00 [1.00,1.00]	1.00 [1.00,1.00]
Intermediate	0.84 [0.74,0.95]	0.95 [0.83,1.09]	0.85 [0.74,0.98]	0.96 [0.83,1.12]	0.84 [0.66,1.06]	0.92 [0.72,1.18]	0.79 [0.71,0.88]	0.92 [0.82,1.04]
High	0.85 [0.73,0.99]	0.98 [0.82,1.17]	0.71 [0.57,0.89]	0.88 [0.68,1.13]	0.75 [0.58,0.96]	0.97 [0.72,1.31]	0.81 [0.72,0.92]	1.03 [0.89,1.18]
p-value	0.015	0.770	0.004	0.592	0.079	0.796	< 0.001	0.120
**Income**								
Low	1.29 [1.07,1.55]	1.24 [1.00,1.54]	1.97 [1.55,2.51]	1.52 [1.16,1.99]	1.72 [1.27,2.33]	1.42 [0.99,2.03]	1.72 [1.47,2.02]	1.05 [0.87,1.27]
Intermediate	1.15 [1.00,1.32]	1.13 [0.97,1.33]	1.47 [1.21,1.81]	1.16 [0.93,1.45]	1.39 [1.13,1.72]	1.21 [0.94,1.54]	1.29 [1.18,1.42]	0.94 [0.84,1.05]
High	1.00 [1.00,1.00]	1.00 [1.00,1.00]	1.00 [1.00,1.00]	1.00 [1.00,1.00]	1.00 [1.00,1.00]	1.00 [1.00,1.00]	1.00 [1.00,1.00]	1.00 [1.00,1.00]
p-value	0.025	0.146	< 0.001	0.004	< 0.001	0.135	< 0.001	0.310
**Region**^**b**^								
South-East	1.10 [1.04,1.16]	1.11 [1.05,1.17]	1.07 [1.01,1.14]	1.08 [1.01,1.15]	0.85 [0.78,0.93]	0.85 [0.78,0.93]	0.95 [0.91,0.98]	0.96 [0.92,1.00]
West	0.93 [0.83,1.04]	0.93 [0.83,1.04]	0.99 [0.88,1.13]	1.01 [0.88,1.15]	1.12 [0.92,1.36]	1.09 [0.89,1.33]	1.41 [1.30,1.53]	1.39 [1.27,1.51]
Mid	1.10 [0.97,1.25]	1.09 [0.95,1.25]	1.21 [1.02,1.42]	1.22 [1.02,1.45]	2.67 [2.22,3.21]	2.82 [2.32,3.43]	0.76 [0.68,0.85]	0.75 [0.67,0.84]
North	0.62 [0.51,0.75]	0.59 [0.49,0.72]	0.55 [0.44,0.70]	0.52 [0.41,0.66]	0.48 [0.31,0.75]	0.48 [0.31,0.75]	1.02 [0.89,1.17]	1.04 [0.89,1.20]
p-value	< 0.001	< 0.001	< 0.001	< 0.001	< 0.001	< 0.001	< 0.001	< 0.001
**Comorbidity**								
No admissions	1.01 [0.75,1.36]	0.97 [0.71,1.32]	0.53 [0.26,1.06]	0.48 [0.23,0.97]	0.74 [0.58,0.96]	0.90 [0.69,1.17]	1.76 [1.55,2.00]	2.00 [1.75,2.29]
0	1.00 [1.00,1.00]	1.00 [1.00,1.00]	1.00 [1.00,1.00]	1.00 [1.00,1.00]	1.00 [1.00,1.00]	1.00 [1.00,1.00]	1.00 [1.00,1.00]	1.00 [1.00,1.00]
1–2	1.21 [1.06,1.39]	1.18 [1.02,1.36]	1.23 [1.07,1.41]	1.28 [1.10,1.49]	1.09 [0.79,1.51]	1.07 [0.75,1.51]	1.65 [1.47,1.85]	1.38 [1.22,1.56]
3+	1.83 [1.42,2.36]	1.67 [1.28,2.18]	1.95 [1.55,2.45]	1.90 [1.48,2.44]	1.94 [1.02,3.68]	1.09 [0.54,2.19]	3.24 [2.52,4.17]	2.31 [1.75,3.06]
p-value	< 0.001	< 0.001	< 0.001	< 0.001	< 0.001	< 0.001	< 0.001	< 0.001

^a^Colorectal, lung and prostate: Localised (A), Regional (B), Metastasis (C), Unknown (D). Breast: Stage I-II (A), Stage III (B), Stage IV (C), Unknown (D)

^b^Compared with Norway (mean)

^*^Odds Ratio [95% c onfidence interval]

### Sex

Male lung cancer patients had a lower odds of not being included in a CPP compared to women (OR = 0.86, 95% CI: 0.75–0.99) (Table 2). No sex difference was observed among colon cancer patients (OR = 1.09, 95% CI: 0.95–1.25), while male rectum cancer patients had a reduced odds of not being included in a CPP compared to women (OR = 0.71, 95% CI: 0.55–0.91).

### Stage

Among colorectal cancer patients diagnosed with a regional stage, 87.1% (84.8% for colon and 93.0% for rectum) were included in a CPP, as compared to 79.5% among patients with a localised tumour (Supplementary Table 2). Similarly, 79.4% of patients with regional prostate cancer were included in a CPP as compared to 62.7% among patients with a localised tumour. Being diagnosed with a metastatic tumour was associated with an increased odds of not being included in a CPP for colon (OR = 1.37, 95% CI: 1.13–1.66), lung (OR = 3.44, 95% CI: 2.72–4.35) and breast (stage IV) cancer (OR = 5.94, 95% CI: 4.20–8.40) (Table 2). For rectum cancer patients, a reduced odds was observed for patients with metastatic tumour (OR = 0.59, 95% CI: 0.41–0.83) (data not shown).

### Region

The proportion of cancer patients included in a CPP varied between the different health regions (p < 0.001). The probability adjusted for case-mix ranged from 81.4% (West) to 86.9% (North) for all cancer sites together (data not shown). The variability between the different regions ranged from 7.7 percentage points (from 80.0% in Mid to 87.7% in North) among colorectal cancer patients to 13.6 percentage points (from 60.0% in West to 73.5% in Mid) for prostate cancer patients (Table 1). For colorectal, lung and breast cancer, the northern region had the highest proportion of patients in a CPP, a difference that remained significant after adjusting for case-mix (Table 2). For breast cancer, patients living in region Mid had the highest odds in 2015–2016 (OR = 2.82 95% CI: 2.32–3.43) of not being included in a CPP when compared to the national average. Patients residing in the Mid region in 2016 had a reduced odds (OR = 0.49, 95% CI: 0.26–0.92) of not being included in a CPP for breast cancer. Prostate cancer patients living in the regions South-East and Mid had a 4 and 34% reduced odds of not being included in a CPP, respectively, while patients living in the West had a 28% increased odds of not being included in a CPP (OR = 1.39, 95%:1.27–1.51). No difference was observed when comparing patients living in the North with the national average (Table 2).

### Income and education

For colorectal and lung cancer patients, there was an increased odds of no inclusion in a CPP when comparing patients in the low to the high income group with the OR = 1.24, 95% CI: 1.00–1.54 and OR = 1.52, 95% CI: 1.16–1.99, respectively. For breast cancer, there was a marginally nonsignificant increase in the odds of no CPP when comparing patients in the low to the high income group (OR = 1.42, 95% CI: 0.99–2.03), and no association was observed for prostate cancer patients (OR = 1.05, 95%CI: 0.87–1.27) (Table 2). After adjusting for household income, level of education was no longer significantly associated with the odds of not being included in a CPP (p = 0.111).

### Marital status

There was an 11% (OR = 0.89, 95% CI: 0.80–0.98) and 19% (OR = 0.81, 95% CI: 0.72–0.92) reduced odds of not being in a CPP if the patient was married compared to being single for prostate and colorectal cancer patients, respectively (Table 2). For lung and breast cancer, no significant associations were seen.

### Comorbidity

The odds of not being included in a CPP, after adjusting for case-mix, were significantly higher when comparing patients with a high level (CCI = 3+), to patients with a low level (CCI = 0) of comorbidity for colorectal (OR = 1.67, 95% CI: 1.28–2.18), lung (OR = 1.90, 95% CI: 1.48–2.44) and prostate (OR = 2.31, 95% CI: 1.75–3.06) cancer patients (Table 2). No hospital admissions prior to diagnosis was associated with an increased odds of not being included in a CPP for rectum cancer (OR = 1.81, 95% CI: 1.12–2.92) and prostate cancer patients (OR = 2.00, 95%CI: 1.75–2.29), while the opposite was seen among lung cancer patients (OR = 0.48, 95% CI: 0.23–0.97). For breast cancer, no association was observed (p = 0.817).

## Discussion

The proportion of cancer patients included in a CPP exceeded the national target of 70% in 2015 for colorectal, lung and breast cancer, while this target was met in 2016 for prostate cancer. In addition to patient characteristics such as age, marital status, income, place of residence and comorbidity, patient’s cancer type and stage at diagnosis were identified as independent predictive factors for not being included in a CPP in Norway in 2015–2016.

The proportion of patients who were included in a CPP in 2015–2016 varied substantially between the different cancer types. The highest rates of inclusion were observed for breast cancer patients, over 90% of whom were referred to a CPP in all regions in Norway from June 2015 onwards. This may be explained by the structure and implementation of breast diagnostic centres, as well as, a national breast cancer screening programme that has existed for over two decades. In addition, breast cancer is the only CPP where patients can also be included after a diagnosis. For prostate cancer, referral rates reached over 70% by January 2016, being the lowest inclusion rate among the cancer sites examined here. This can partly be explained by the fact that a low, but still elevated PSA-test, would not be enough for further examinations for prostate cancer since PSA screening has not been implemented in Norway. Another plausible explanation is that some of the patients presenting themselves with lower urinary tract symptoms (LUTS) will have an elevated PSA detected, a biopsy performed and finally being diagnosed with prostate cancer as part of the examination of the LUTS. In addition, some of the prostate cancer patients are diagnosed based on random findings at other procedures like transurethral resection of the prostate (TURP) and cystectomy, and thus will not be included in a CPP. Unlike breast cancer where the symptoms are better defined and more easily detected, the lower rates of CPP referral for prostate cancer may also be partially explained by the nature of the disease and the discretion of the urologist.

The CPPs were implemented in Norway without any extra governmental funding or technological support, but a marked increase in the proportion of patients included in a CPP was observed for all cancer sites from 2015 to 2016. Plausible explanations for this increase include the increased attention to CPPs among policy makers, health personnel at hospitals and regular monitoring. A gradual implementation of CPP coordinators, who are responsible for the reporting of CPPs to the NPR, may also have been a contributing factor.

A patient’s age was identified as a predictor of not being included in CPP in Norway. For colorectal cancer patients the standardised criteria for inclusion in the CPP are restricted to patients over 40 years of age. Hence, the symptoms that cause reasonable suspicion of colorectal cancer for patients under 40 years are less specific, thus it may be more difficult to identify these. For lung cancer increasing age was identified as a positive predictor of not being included in a CPP. Older patients are more likely to have their cancer discovered as part of admissions due to other causes (e.g., COPD). A higher odds of not being included in a CPP among elderly lung cancer patients could therefore be a result of residual confounding of comorbidity. However, a sensitivity analysis showed small changes in the age effect when adjusting for comorbidity. In this paper, patients diagnosed only by radiological examination were included. For the lung cancer patients whose diagnoses were radiologically verified the median age was 10 years higher (80 versus 70 years) and the proportion included in a CPP was almost 20 percentage points lower (63.9% versus 83.5%), when compared to patients with a morphologically verified diagnosis. This can explain some of the observed age effect among the oldest lung cancer patients. Similarly in Denmark, differences in the age distribution for all cancers combined exist when comparing CPP with no CPP patients and the highest odds of inclusion to CPP among colorectal cancer patients were observed in the age group 55–64 [30, 31].

Stage at the time of diagnosis was found to be associated with not being included in a CPP. Around 15–25% of all colon cancer patients are hospitalised due to acute symptoms of obstruction, perforation or bleeding and diagnosed at the time of acute surgery or other intervention [32]. These patients are not included in a CPP and explain the results of higher odds of not being included in a CPP among colon cancer patients with metastatic tumour. A reduced odds of not being included in a CPP was observed among metastatic rectum cancer patients, however we have no obvious explanation of these results. For both colorectal and prostate cancer, the lowest odds of not being included in a CPP were observed among patients with regional spread. Early stage colorectal cancers may be diagnosed incidentally by colonoscopy for other symptoms or in a screening programme. Registration into CPP may be lower in this setting. For prostate cancer, most patients with a low risk localised tumour, are not supposed to undergo any radical treatment, but instead undergo active surveillance. The association between minimally elevated PSA and low risk disease is however not apparent, but PSA-level may have influenced CPP referral. Thus, the PSA level is important for how the risk of prostate cancer and its severity is evaluated and will also affect if patients are included in a CPP. As the severity of lung cancer increases, the odds of not being included in a CPP increased. Patients with an early-stage disease need many different examinations in order to confirm the diagnosis, as well as, deciding what treatment will be the most beneficial. On the other hand, patients with metastasis need only a biopsy of the lung tissue to confirm the diagnosis and decide the potential treatment. The increased odds of not being included in a CPP among the small group of stage IV breast cancer patients can be explained by differences in the diagnostic and treatment “pathway”. This subgroup of breast cancer patients are not primarily identified by a breast tumour which initiates the CPP, but by disseminated disease secondarily diagnosed during hospitalisation. In Denmark, Jensen et al. showed that patients being referred to a CPP had a 20% reduced odds of being diagnosed with a localised tumour when compared to no-CPP patients [33].

After adjusting for case-mix, low household income was associated with an increased odds of not being included in a CPP among colorectal, lung and breast cancer patients. Studies have shown that a patient’s socioeconomic status, represented through household or personal income, education or other measures, is associated with an increased likelihood of access to different aspects of cancer care [34, 35]. Patients with a high household income can be seen as a group who are well-informed about health care options, more demanding in their meeting with the clinicians, and may want a more active role in the decision-making process. For prostate cancer, no association with income was observed. One possible explanation could be that high income prostate cancer patients are using private health care services to reduce the waiting time in the public health care system. A Danish study from 2019 on ovarian cancer showed a 47% increased prevalence ratio in CPP for patients with high socioeconomic status (measured through educational level) [36]. A single hospital trust in England found no association in colorectal, lung, prostate and ovarian cancer between their socioeconomic position and the selection of patients to urgent referral [37].

This study has some limitations. First, the period included in this study is the first 2 years after implementation of CPPs in Norway. In the beginning there may have been start-up problems related to IT-systems, registration practice at the different hospitals, and more uncertainty around the whole concept of CPPs. Hence, the results may not perfectly represent the current situation. Second, there is no available information about the patient pathway from the GP. By having this information, a GP’s propensity to refer a patient to CPPs could help explain differences in inclusion to CPP both at a regional and national level. By using complete information about which patients were included in a CPP, this study provides unique and important information about use of CPPs in Norway during the first years after implementation. The study’s population-based design and the use of national, comprehensive, high quality data provide results that are widely representative.

## Conclusions

This study showed that even though the target of 70% included in a cancer patient pathway was met quite quickly after implementation in Norway, patients with a prostate cancer diagnosis, older age, high level of comorbidity, or low income were identified as having a high risk of not being included in a CPP. The variation in these factors may be a residue of a quick implementation of CPPs in Norway, and therefore these should be monitored to ensure that there is equal access for all cancer patients.

## Supplementary information

**Additional file 1 : Supplementary Table 1:** Patient characteristics for all colorectal, lung, breast and prostate cancer patients, and for those who were and were not included in a cancer patient pathway.

**Additional file 2 : Supplementary Table 2:** Probability of being included in a cancer patient pathway after adjusting for case-mix (year of diagnosis, age group, sex, tumour stage, marital status, education, income, region of residence and comorbidity) for colorectal, lung, breast and prostate cancer patients diagnosed in 2015–2016.

## Data Availability

The data that support the findings of this study are available upon request from the Cancer Registry of Norway, The Norwegian Patient Registry and Statistics Norway.

## Abbreviations

CCI: Charlson comorbidity index
CI: Confidence interval
CPP: Cancer patient pathway
CRN: Cancer registry of Norway
GP: General practitioner
ICD-10: International classification of diseases
LUTS: Lower urinary tract symptoms
NPR: Norwegian patient registry
OR: Odds Ratio
PSA: Prostate-specific antigen
SES: Socioeconomic status
TURP: Transurethral resection of the prostate
